# Supplementary material for: Various plasmid strategies limit the effect of bacterial restriction–modification systems against conjugation
Source: Nucleic Acids Res. 2024 Oct 16;52(21):12976–86. doi: 10.1093/nar/gkae896 (PMC11602122; doi:10.1093/nar/gkae896)
Supplement: gkae896_Supplemental_Files [file gkae896_supplemental_files.zip › RM_plasmids_revision_SI.pdf]

plasmid	size (bp)	MOB	MPF	Replicon	AMR genes
R388	33913	-	-	IncW	AAC(6')-Ib7;dfrB2;qacEdelta1;sul1
R6K	39872	MOBP	typeT	IncX2	APH(3'')-Ib;APH(6)-Id;TEM-181
pCU1	41131	MOBF	typeT	IncN	AAC(6')-Ib7;ANT(3'')-IIa;OXA-2;OXA-2;qacEdelta1;sul1
RIP113	42161	MOBF	typeT	IncN	tet(A)
pCF12	43704	MOBP	typeT	IncX3	QnrS1;SHV-66
RK2	60096	MOBP	typeT	IncP1	APH(3')-Ib;TEM-181;tet(A)
pOXA48	61881	MOBP	typeI	IncL/M	OXA-48
pB10	64508	MOBP	typeT	IncP1	AAC(6')-Ib7;APH(3'')-Ib;APH(6)-Id;OXA-2;qacEdelta1;sul1;tet(A)
pCT	93629	MOBP	typeI	IncB/O/K/Z	CTX-M-14
R1	97566	MOBF	typeF	IncFII	ANT(3'')-IIa;APH(3')-Ia;TEM-181;catI;qacEdelta1;sul1
pESBL283	110137	MOBP	typeI	IncI1	AAC(6')-Ib7;ANT(3'')-IIa;CTX-M-1;dfrA1;qacEdelta1;sul1;sul2
pRK100	142357	MOBF	typeF	IncFIB;IncFII	TEM-181;tet(A)
pKAZ3	147957	MOBH	typeF	IncA/C2	AAC(6')-Ib7;QnrVC1;VEB-9;dfrA1;dfrA23;qacEdelta1;qacEdelta1;su

Table S1: wildtype conjugative plasmids used in this study.

**RM system**	**type**	**strain**	**plasmid**	**recognition sequence** (5′- -3′)
EcoAI	IB	NK354	NA	GAGNNNNNNNGTCA
EcoBI	IA	WA251	NA	TGANNNNNNNNTGCT
EcoKI	IA	MG1655	NA	AACNNNNNNGTGC
EcoR124I	IC	NK402	NA	GAANNNNNNRTCG
EcoRI	IIP	MG1655 ΔhsdS::KnR	pMB1 ori, bla	GAATTC
EcoRII	IIEP	MG1655 ΔhsdS::KnR	pMB1 ori, bla	CCWGG
EcoRV	IIP	MG1655 ΔhsdS::KnR	pMB1 ori, bla	GATATC
EcoVIII	IIP	MG1655 ΔhsdS::KnR	pMB1 ori, bla	AAGCTT
EcoP1	III	MG1655 ΔhsdS::KnR	p15A ori, cat	AGACC
EcoP15I	III	MG1655 ΔhsdS::KnR	p15A ori, cat	CAGCAG

Table S2: RM systems used in this study. R stands for A or G; W stands for A or T.

number	Name	Sequence
TD_RM1	H1_dcm_p1	TGTAATTATGTTAACCTGTCGGCCATCTCAGATGGCCGGTGAAATCTATGGTGTAGGCTGGAGCTGCTTC
TD_RM2	H2_dcm_p2	TCGCGCGCATATTTTTGCTGCGAGTGGCCTTATCGTGAACGTCGGCCATGATGGGAATTAGCCATGGTCC
TD_RM3	dcm - 1,854,543 F	TTTTCATCCGCTGGCTGAG
TD_RM4	dcm - 1,856,105 R	CGCTTCTCTATCGCCGTATC
TD_RM5	H1_pB10_klcA_p1	CTTGAAGGCCGGGCATTTCGCCCAGGTCAACTACCGGAGAAAGTCCGATGGTGTAGGCTGGAGCTGCTTC
TD_RM6	H2_pB10_klcA_p2	GCCCCGGCCGAAGCCGGGGCCGCCCCTCATCAGTCAATAGCCCGGTAGATATGGGAATTAGCCATGGTCC
TD_RM7	H1_pB10_klcB_p1	CGCGACGTGATCGCGTGGCGGCGTGCTGACACTTGAGGGGCCGGCCGATGGTGTAGGCTGGAGCTGCTTC
TD_RM8	H2_pB10_klcB_p2	CGTCGTTCGTCATTCGTTCGTTCTCCTGGTCAAGTGATAAGCCGCAACTGATGGGAATTAGCCATGGTCC
TD_RM9	pB10_klcA - 40,987 F	GTTGTTCCTGGTCATGGTCG
TD_RM10	pB10_klcA - 41,599 R	AGGGAAAGGGCAAGGTTTAG
TD_RM11	pB10_klcB - 39,515 F	CACTCCAGCCGGATATTGG

TD_RM12	pB10_klcB - 40,868 R	GGTTCAGCTCGGGATTCTG
TD_RM13	H1_pKAZ3_dam1_p1	TTCGTTTCTACCTCTTGAATGTTTCGTCAGAACAAACTGGAGCGCTTATGGTGTAGGCTGGAGCTGCTTC
TD_RM14	H2_pKAZ3_dam1_p2	ATGCAAAACCCGCCAATCGGCGGGTTTTTTATGCTGCCACGGTAGCGGCATGGGAATTAGCCATGGTCC
TD_RM15	pKAZ3_dam1 - 22,794 F	AGGTCTTCGTTCTTGAACCG
TD_RM16	pKAZ3_dam1 - 23,647 R	TGCCCCGACTTTATCAAGAC
TD_RM17	H1_pKAZ3_dcm3_p1	TTCCCGATTGGGACGGTAGTGGCCTCTCAACCGAAAGGAGAGAGCTCATGGTGTAGGCTGGAGCTGCTTC
TD_RM18	H2_pKAZ3_dcm3 p2	AGGCCTTTCGGCATTGCCCCGGTGAGTTACTAATGAACCAAAGATGAAGTATGGGAATTAGCCATGGTCC
TD_RM19	pKAZ3_dcm3 - 80,458 F	ACGACTCTGACGAAACGAAG
TD_RM20	pKAZ3_dcm3- 82,422 R	CTGGCAATGCAGTGTTGAAG
TD_RM21	H1_vcrx089	CCGCCCGGTTCGGGACGTGGTGCGCCTGTCACAATAGGAGCGCACCTATGGTGTAGGCTGGAGCTGCTTC
TD_RM22	H2_Vxrx090	CCCTTTCGGGGAGCAAGCCCCCGTAAGGGTTAAAAAAAGTGATCCTCTTGATGGGAATTAGCCATGGTCC
TD_RM23	H1_Vcrx093	CTCCCCCGCTGGGGGAGATCTCCCTCCTAACCATTGGAGAGAGGTCCATGGTGTAGGCTGGAGCTGCTTC
TD_RM24	H2_Vcrx093	CCAAGGGAAAGCGGGGGAAAATCCCCCACTCACCAGTAGAACGTCTCTACATGGGAATTAGCCATGGTCC
TD_RM25	pKAZ3 - 66,670 F vcrx08990	TCTTCGGAGAGCGAAAAGTG
TD_RM26	pKAZ3 - 68,800 R vcrx08990	CAGAACCGAGATTACCGACC
TD_RM27	pKAZ3_dvcrx093F	CCTTCGTGACAAGGAGATGG
TD_RM28	pKAZ3_dvcrx093R	TGTTCACCCTTGTTACACCG
TD_RM29	H1_ardB_RIP113	TACCGCCGCCCGAAAAATTGCCGGTATCGGCTTAAAGAGGAAGTATCATGGTGTAGGCTGGAGCTGCTTC
TD_RM30	H2_arcB_RIP113	AAGGCGGGCTTTGCCCGCCTGGTTATCAGTTAATCAATGGCACGATAAATATGGGAATTAGCCATGGTCC
TD_RM31	RIP113_ardB - 1,123 F	CAGACGGTAGGGTGTTGAAG
TD_RM32	RIP113_ardB - 1,781 R	CTTCCATGCCCTGACTGG
TD_RM33	H1_RIP113_umuC	CCATAACAGCGATACTGTATAAATAAACAGTTATTTGGAAGATCGCTATGGTGTAGGCTGGAGCTGCTTC
TD_RM34	H2_RIP113_umuD	GTTAACCGGCCCGGGTACGGCGCCGGTAATTATTTGATGGTGGCTATTGGATGGGAATTAGCCATGGTCC
TD_RM35	RIP113umuC - 1,934 F	ATATAGCGTTCGTTGGCAGC
TD_RM36	RIP113umuD - 3,808 R	GAAACCGTGCCGAAACTTG

Table S3: primers used in this study.

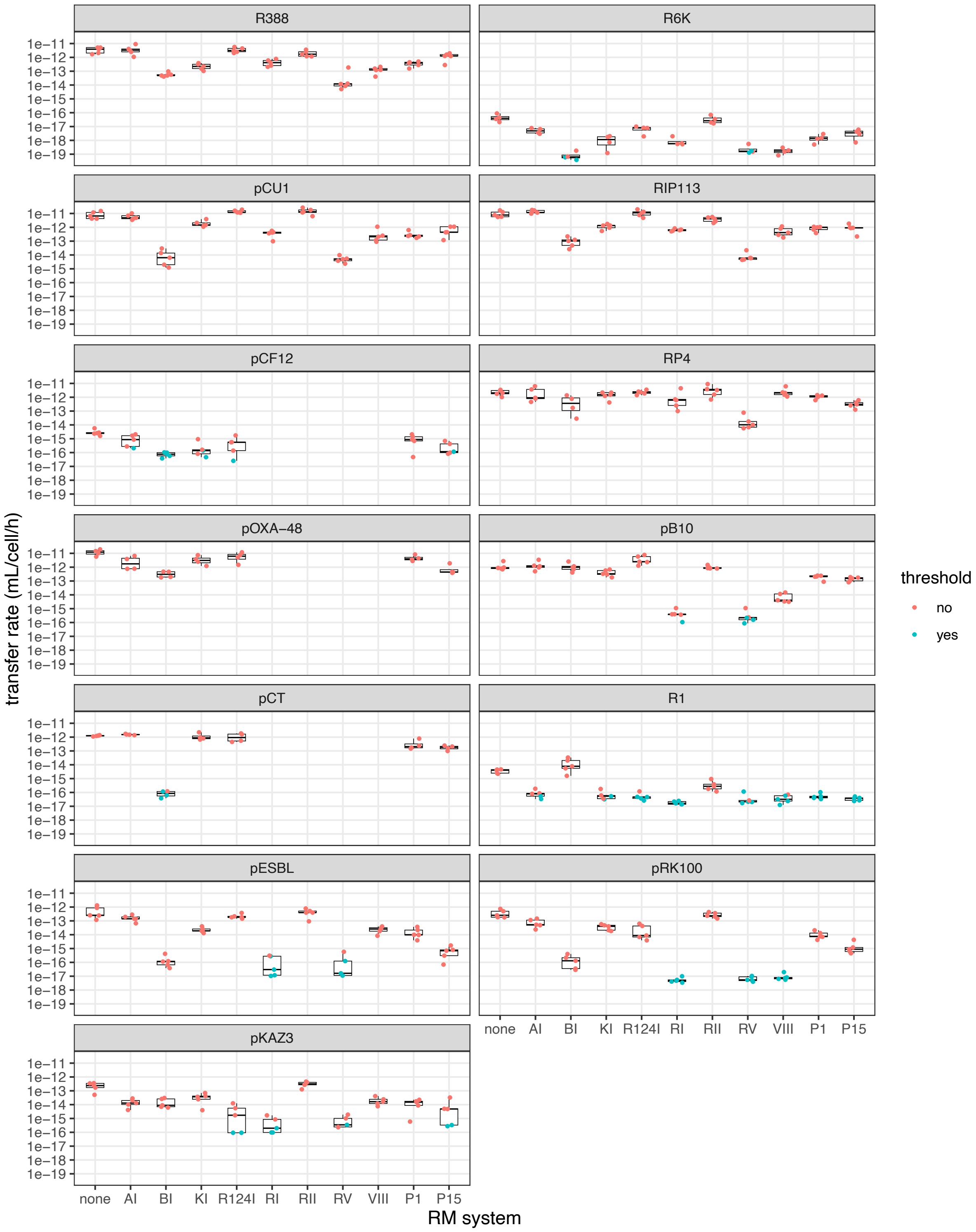

Figure S1: transfer efficiency of wildtype plasmids. Each facet shows transfer efficiency for one of 13 wildtype plasmids, as a function of the *E. coli* RM system present in the recipient strain. The centre value of the boxplots shows the median, boxes the first and third quartile, and whiskers represent 1.5 times the interquartile range. Individual replicates are shown as dots (n≥4); red dots indicate that transconjugant colonies were counted, whereas blue dots indicate that no transconjugants were observed and the threshold transfer efficiency is shown instead.

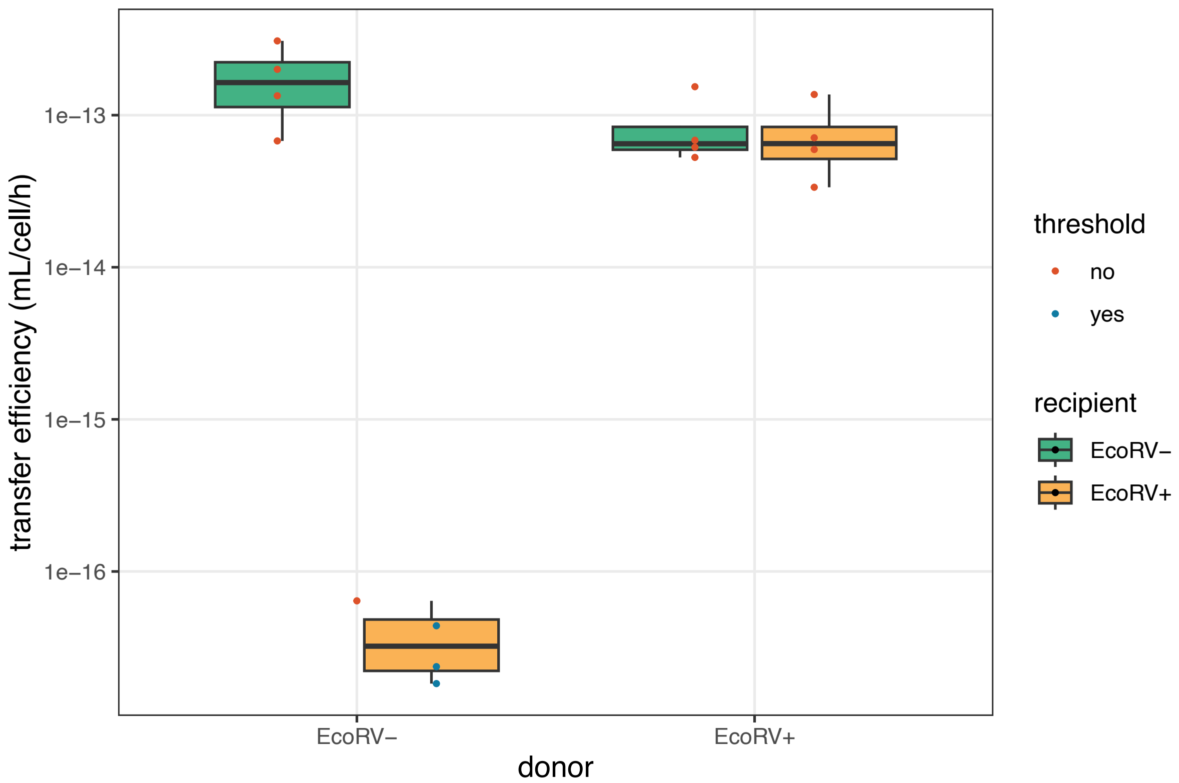

Figure S2: effect of RM system presence in the donor on conjugation for the pRK100-EcoRV pair. Transfer efficiency of pRK100 plasmid is shown from an EcoRV- or EcoRV+ donor strain to an EcoRV- or EcoRV+ recipient strain. The centre value of the boxplots shows the median, boxes the first and third quartile, and whiskers represent 1.5 times the interquartile range. Individual replicates are shown as dots (n=4). Red dots indicate that transconjugant colonies were counted, whereas blue dots indicate that no transconjugants were observed and the threshold transfer efficiency is shown instead.

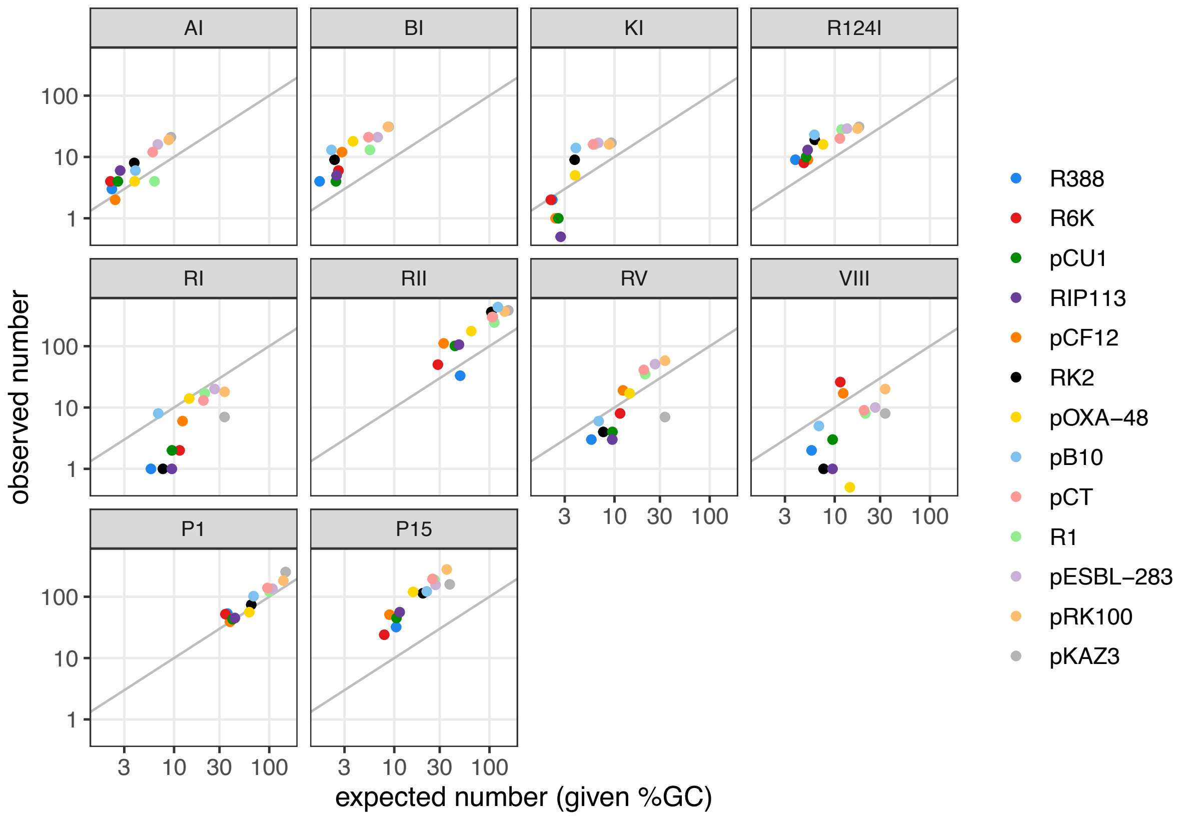

Figure S3: expected and measured number of RM recognition sites, ordered by RM system. The expected number of recognition sites is calculated by taking into account the base composition of the recognition sequence, and the overall %GC of the plasmid considered, using the following formula: $0.5\ (1\text{-}\%GC)^{n(\ A+T)} * 0.5\ (\%GC)^{n(\ G+C)}$*sequence length. Observed values are shown as a function of expected values, as a log scale.

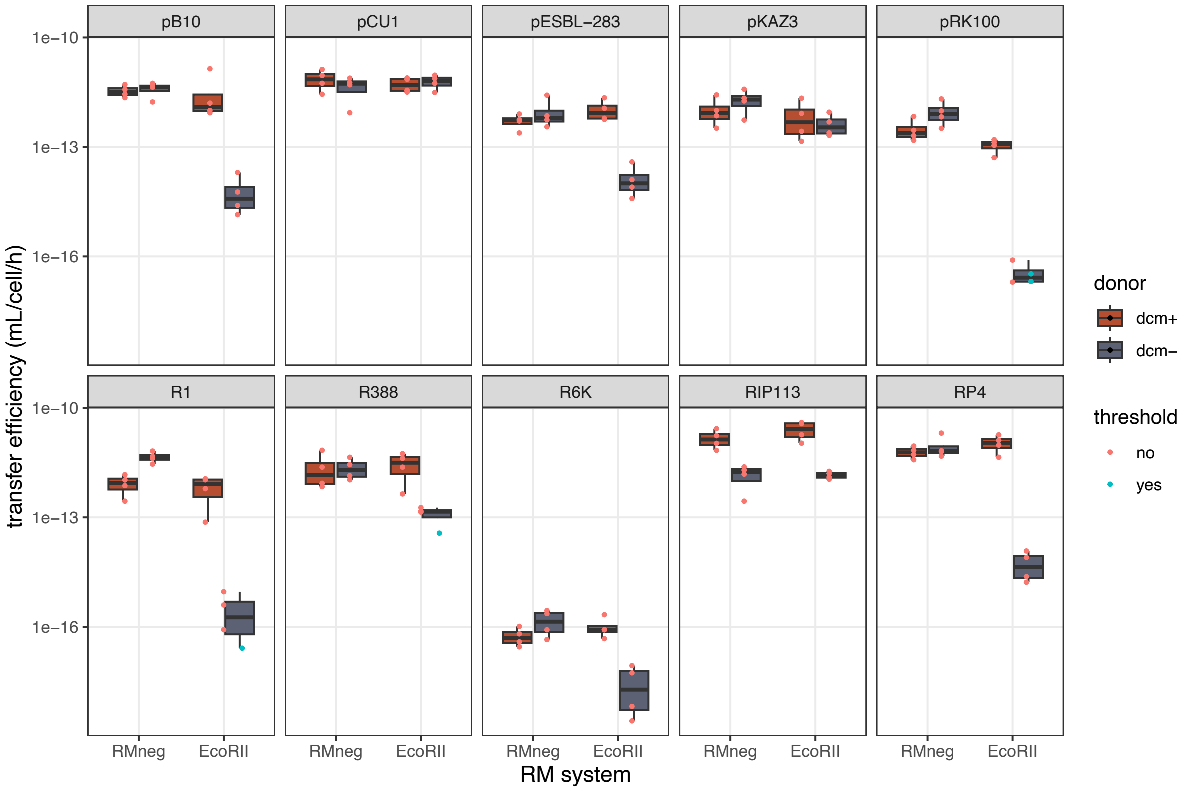

Figure S4: transfer efficiency of wildtype plasmids from *dcm+* and *dcm-* donors. Each facet shows transfer efficiency for one of 10 wildtype plasmids, as a function of the *E. coli* RM system present in the recipient strain (none or EcoRII). The centre value of the boxplots shows the median, boxes the first and third quartile, and whiskers represent 1.5 times the interquartile range. Individual replicates (n = 4) are shown as dots; red dots indicate that transconjugant colonies were counted, whereas blue dots indicate that no transconjugants were observed and the threshold transfer efficiency is shown instead.

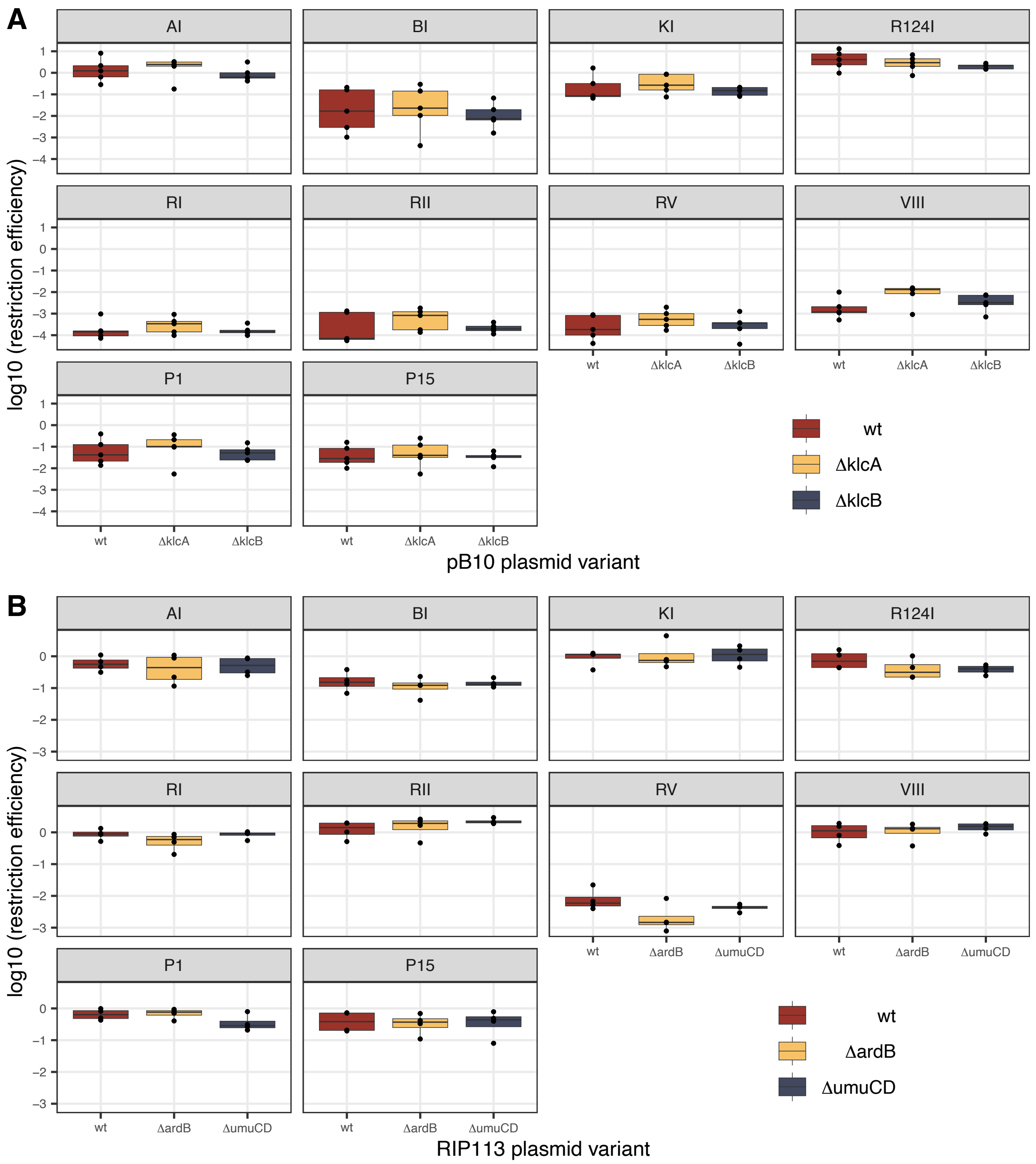

Figure S5: effect of additional plasmid anti-restriction functions on restriction efficiency. Log-transformed restriction efficiency for 10 *E. coli* RM systems is shown from a *dcm+* donor strain for wild-type and mutant pB10 (A) and wild-type and mutant RIP113 (B). The centre value of the boxplots shows the median, boxes the first and third quartile, and whiskers represent 1.5 times the interquartile range. Individual replicates are shown as dots (n≥4). Mutant plasmids showed no significant difference from wild-type plasmids in restriction efficiency in any RM treatment after Bonferroni correction (all $p > 0.05$).

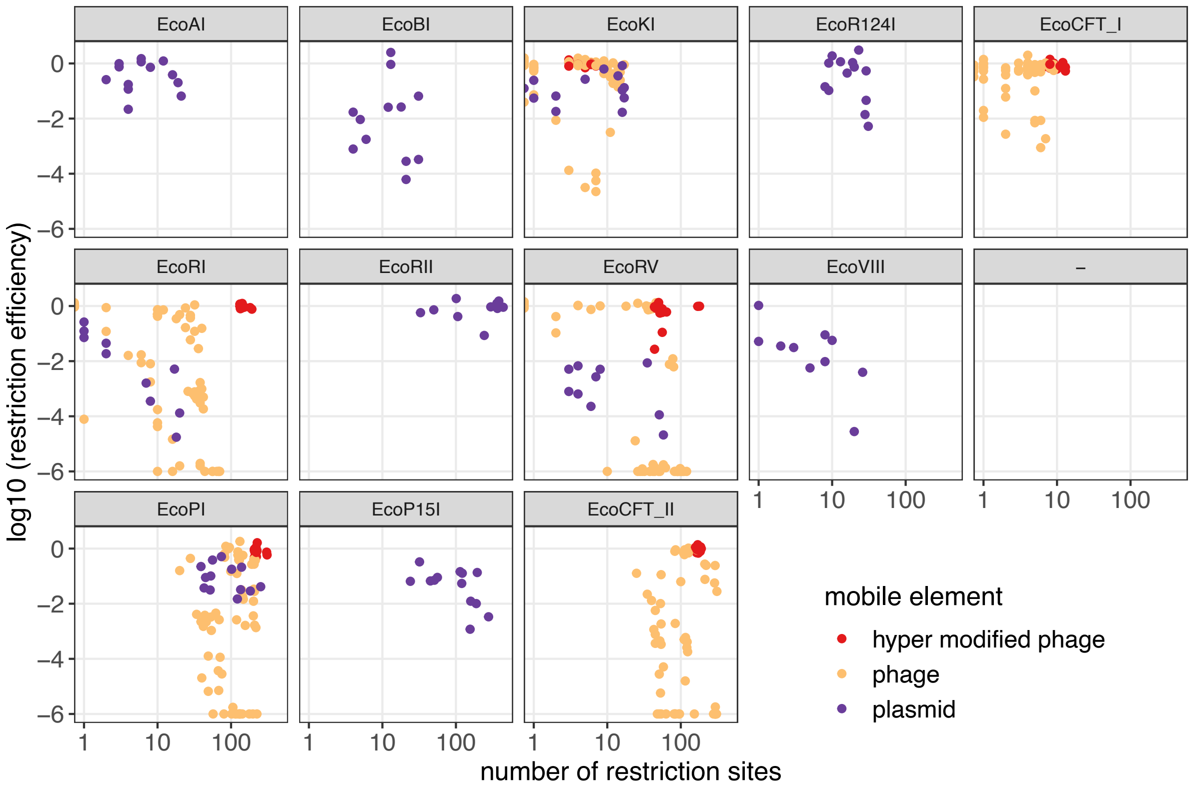

Figure S6: comparison of restriction efficiency against conjugative plasmids and phages. Mean values of restriction efficiency data presented in Fig. 2 for conjugative plasmids and mean values of restriction efficiency calculated from EOP (efficiency of plating) data in [1] (Maffei et al.) using the BASEL collection of *E. coli* phages, are shown as a function of RM recognition site number on each element. Plasmid data are shown in violet; phage data are shown in orange, except phages from the Tevenvirinae (T-even phages), which are known to be hyper-modified and shown in red. A value of 10^{-6} was added to the phage EOP data as an estimated detection threshold limit similar to the threshold applied to our conjugation data.

[1] Maffei et al., 'Systematic Exploration of Escherichia Coli Phage–Host Interactions with the BASEL Phage Collection'.
